# Role of Histamine and Related Signaling in Kaposi’s Sarcoma-Associated Herpesvirus Pathogenesis and Oncogenesis

**DOI:** 10.3390/v15041011

**Published:** 2023-04-20

**Authors:** Jungang Chen, Jiao Song, Karlie Plaisance-Bonstaff, Shengyu Mu, Steven R. Post, Lu Dai, Zhiqiang Qin

**Affiliations:** 1 Department of Pathology, Winthrop P. Rockefeller Cancer Institute, University of Arkansas for Medical Sciences, Little Rock, AR 72205, USA; 2 Department of Medicine, Louisiana State University Health Sciences Center, Louisiana Cancer Research Center, New Orleans, LA 70112, USA; 3 Department of Pharmacology & Toxicology, University of Arkansas for Medical Sciences, Little Rock, AR 72205, USA

**Keywords:** KSHV, Kaposi’s Sarcoma, histamine, histamine receptor

## Abstract

Although Kaposi’s sarcoma-associated herpesvirus (KSHV) has been reported to cause several human cancers including Kaposi’s sarcoma (KS) and primary effusion lymphoma (PEL), the mechanisms of KSHV-induced tumorigenesis, especially virus–host interaction network, are still not completely understood, which therefore hinders the development of effective therapies. Histamine, together with its receptors, plays an important role in various allergic diseases by regulating different inflammation and immune responses. Our previous data showed that antagonists targeting histamine receptors effectively repressed KSHV lytic replication. In the current study, we determined that histamine treatment increased cell proliferation and anchorage-independent growth abilities of KSHV-infected cells. Furthermore, histamine treatment affected the expression of some inflammatory factors from KSHV-infected cells. For clinical relevance, several histamine receptors were highly expressed in AIDS-KS tissues when compared to normal skin tissues. We determined that histamine treatment promoted KSHV-infected lymphoma progression in immunocompromised mice models. Therefore, besides viral replication, our data indicate that the histamine and related signaling are also involved in other functions of KSHV pathogenesis and oncogenesis.

## 1. Introduction

Kaposi’s Sarcoma (KS) is caused by Kaposi’s sarcoma-associated herpesvirus (KSHV) infection. The major feature of KS is its composition, which consists of spindle cells (the tumor cell with endothelial derivatives), a proliferation of abnormal and leaky vessels, as well as extravasated red blood cells with hemosiderin deposits [1]. A prominent inflammatory infiltrate is often observed and reported in the development of these lesions, especially at the early stage. KS has different pathologic stages (early stage—“patch”; middle stage—“plaque”; late stage—“nodule”), while individual patients usually display different types of lesions. However, AIDS-associated KS (AIDS-KS) can display more aggressively, affecting skin, lymph nodes and visceral organs in patients. Despite the reduced incidence of KS due to combined Antiretroviral Therapy (cART) in HIV+ patients, KS still remains one of the most common AIDS-associated malignancies [2]. Further, a longitudinal study conducted among solid organ transplant recipients in US reported 15% of KSHV seropositivity in this population [3]. Transplant recipients with primary KSHV infection after the transplantation were reported to have a relatively high risk of developing KS [4,5]. However, the mechanisms of KSHV-induced tumorigenesis, especially the complicated virus–host interaction, are still not completely understood, which hinders the development of effective therapies.

Histamine is mainly produced and released by mast cells (MCs), acting as a bivalent regulator for cancer development [6]. Histamine, together with its receptors, plays an important role in various allergic diseases by regulating different inflammation and immune responses [7]. Interestingly, MCs were recently reported to be abundant within KS lesions, and MC granule contents were determined to be elevated in plasma from KS patients [8]. Furthermore, our recent data confirmed that antagonists targeting histamine receptors effectively repressed KSHV lytic replication from induced iSLK.219 or KSHV+ primary effusion lymphoma (PEL) cells [9]. Using the samples from a cohort of HIV+ patients, our results confirmed that the KSHV+ group has much higher levels of histamine in the samples of plasma and saliva than those from the KSHV− group [9]. These data together indicate MC-secreted products, especially histamine, as important components of KS/PEL tumor microenvironment, as well as the important role of histamine and related signaling in the regulation of KSHV lytic replication. In the current study, we continue to explore the functional role of histamine and histamine receptors in KSHV pathogenesis and oncogenesis.

## 2. Materials and Methods

### 2.1. Cell Culture, Reagents and Infection Protocols

KSHV+ PEL cell line BCBL-1 was kindly provided by Dr. Dean Kedes (University of Virginia, Charlottesville, USA) and cultured as described previously [9]. Primary human umbilical vein endothelial cells (HUVEC) were purchased from American Type Culture Collection (ATCC, Manassas, VA, USA) and cultured as recommended by the manufacturer. Human iSLK.219 cells (Dox-inducible SLK carrying rKSHV.219) were latently infected with a recombinant rKSHV.219 virus and contained a doxycycline (Dox)-inducible RTA [10]. KSHV long-term-infected telomerase-immortalized HUVEC (TIVE-LTC) cells were kindly provided by Dr. Rolf Renne (University of Florida, Gainesville, USA) and cultured as previously described [11]. To obtain KSHV for the infection experiments, BCBL-1 cells were incubated with valproic acid for 4–6 days, and KSHV was purified from the culture supernatant by ultracentrifugation at 20,000× *g* for 3 h, 4 °C. The infectious titers were determined as described previously [9].

### 2.2. Cell Proliferation and Soft Agar Assays

Cell proliferation was measured using the WST-1 Assay (Roche, Indianapolis, IN, USA). Briefly, after compound treatments, 10 μL/well of chemical reagent, WST-1 (4-[3-(4-Iodophenyl)-2-(4-nitro-phenyl)-2H-5-tetrazolio]-1,3-benzene disulfonate), was added into 96-well microplate for a 3 h incubation at 37 °C. The absorbance of samples was measured by using a microplate reader at 490 nm. The anchorage-independent growth abilities of KSHV+ tumor cells were assessed using soft agar assays as described previously [12].

### 2.3. RT-qPCR

Total RNA was isolated using the RNeasy Mini kit (Qiagen, Germantown, MD, USA), and cDNA was synthesized using a SuperScript III First-Strand Synthesis SuperMix Kit (Invitrogen, Waltham, MA, USA). Primers used for amplification of target genes are listed in Table S1. Amplification was carried out using an iCycler IQ Real-Time PCR Detection System, and cycle threshold (Ct) values were tabulated in duplicate for each gene of interest in each experiment. We used mean Ct values tabulated for each gene, as well as paired Ct values for β-actin as a loading control.

### 2.4. Western Blot

Total cell lysates (20 µg) were resolved by 10% SDS-PAGE, transferred to nitrocellulose membranes and immunoblotted with antibodies to IL-6, IFIT1 and Tubulin (Cell Signaling, Danvers, MA, USA). Immunoreactive bands were identified using an enhanced chemiluminescence reaction (Perkin–Elmer, Waltham, MA, USA) and visualized by autoradiography.

### 2.5. KS Tumor Tissues from HIV+ Patients and Immunohistochemistry

KS tissues from the HIV+ patient cohort were kindly provided by the Louisiana State University Health Sciences Center (LSUHSC) HIV Outpatient (HOP) Clinic and Biospecimens Bank, New Orleans, LA, USA. Immunohistochemistry (IHC) was performed as described previously [13]. The antibody for KSHV major latent protein LANA was purchased from Advanced Biotechnologies (Eldersburg, MD, USA). The antibodies for HRH1-HRH4 proteins were purchased from Abcam (Waltham, MA, USA) and used as recommended by the manufacturers.

### 2.6. PEL Xenograft Models

A total of 1 × 10^7^ BCBL-1 cells in 200 µL RMPI-1640 without FBS were injected intraperitoneally into NOD/SCID mice, 6–8-week-old, male (purchased from Jackson Laboratory, Ellsworth, ME, USA). Then, the mice were randomized into different groups as described previously [14]. The histamine or vehicle control was administered initially at 72 h after tumor cell injections, and continued once daily, 2 days per week for 4 weeks. Weights were recorded weekly as a surrogate measure of tumor progression. All protocols were approved by the University of Arkansas for Medical Sciences Animal Care and Use Committee, Little Rock, AR, USA, in accordance with national guidelines.

### 2.7. Statistical Analysis

Significance for differences between the experimental and control groups (with no fewer than 3 experiments per group performed) was determined by using the two-tailed Student’s *t*-test (Excel 2016, Microsoft, Redmond, WA, USA), and *p* values < 0.05 or < 0.01 were considered significant or highly significant, respectively.

## 3. Results

**Figure 1:**
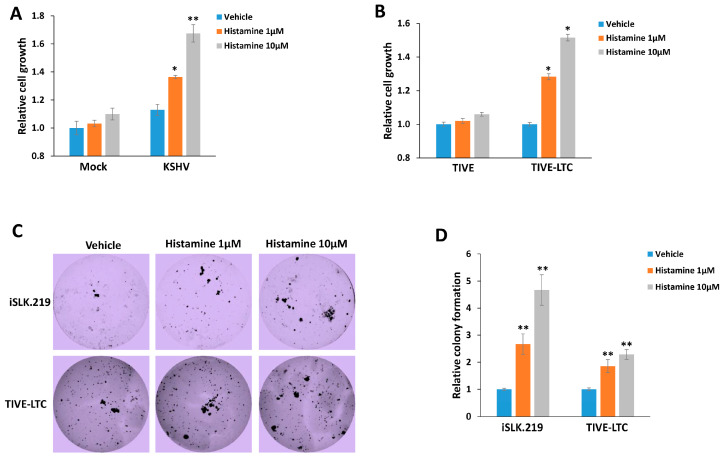
Histamine treatment promotes the growth of KSHV-infected cells. (**A**,**B**) HUVEC with or without KSHV infection ((**A**), MOI~3) or TIVE and KSHV long-term-infected TIVE-LTC (**B**) were treated with indicated concentrations of histamine for 48 h; then, the cell proliferation was measured using the WST-1 assays. (**C**,**D**) The anchorage-independent growth abilities of iSLK.219 and TIVE-LTC were determined using the soft agar assays. Error bars represent the S.D. for three independent experiments. * = *p* < 0.05; ** = *p* < 0.01 (vs. the vehicle groups).

Our recent data showed that the histamine levels in plasma from the KSHV+ group were significantly higher (about 1–4 µM) than those from the KSHV− group of HIV− infected patients [9]. Therefore, here, we used 1–10 µM of exogenous histamine for our in vitro experiments, which represent the pathophysiological concentrations within KSHV/HIV-infected patients. We first compared the effects of exogenous histamine on the growth of HUVEC with or without KSHV infection. Our results showed that this dose range of histamine only slightly increased non-infected cells growth, while both 1 and 10 µM of histamine significantly increased KSHV-infected cells growth at ~30–70% (Figure 1A). This dose range of histamine also significantly increased the growth of TIVE-LTC, a long-term KSHV-infected immortalized endothelial cell line, but not control TIVE cells (Figure 1B). Next, we examined the effects of histamine on the anchorage-independent growth ability of KSHV-infected immortalized cells using the soft agar assays. We determined that exogenous histamine treatment greatly increased the rate of colony formation from both iSLK.219 and TIVE-LTC in a dose-dependent manner (a ~2–5 fold increase) when compared to the vehicle groups (Figure 1C,D). These data indicate that histamine may act as an important component of host microenvironment to facilitate KSHV-induced cell proliferation and tumorigenesis.

**Figure 2:**
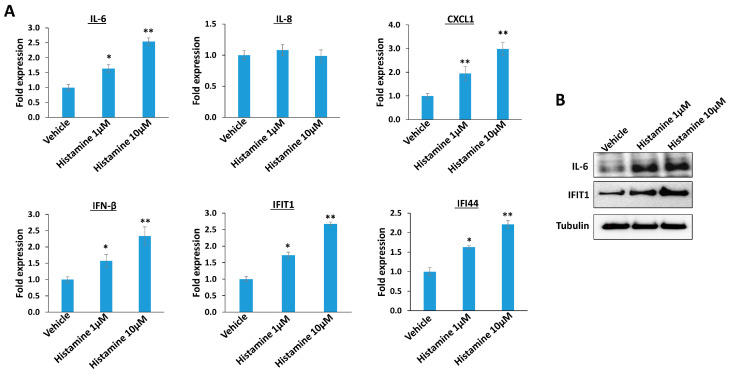
The impacts of inflammatory factors by histamine treatment. (**A**) TIVE-LTC were treated with indicated concentrations of histamine for 48 h; then, gene transcripts were quantified by using RT-qPCR. Error bars represent the S.D. for three independent experiments. * = *p* < 0.05; ** = *p* < 0.01 (vs. the vehicle groups). (**B**) Protein expression was measured using Western blot.

Since histamine has a central role as a mediator in the inflammatory response, we are interested to determine its impacts on other host inflammatory factors which have been reported during KSHV infection [15,16,17]. By using RT-qPCR, we discovered that histamine treatment increased IL-6 (Interleukin 6, one of major pro-inflammatory cytokines) and CXCL1 (CXC motif chemokine ligand 1, one of major inflammatory chemokines) content, but not that of IL-8 from TIVE-LTC (Figure 2A). In addition, histamine treatment increased IFN-β (Interferon-β) as well as IFIT1 (Interferon induced protein with tetratricopeptide repeats 1) and IFI44 (Interferon induced protein 44) content, two of interferon-stimulated genes (ISGs) from TIVE-LTC. By using Western blot, we confirmed that histamine treatment upregulated the expression of these inflammatory factors such as IL-6 and IFIT1 from TIVE-LTC (Figure 2B). Thus, we suppose that histamine may mediate inflammatory response to KSHV infection through regulation of different host factors. In fact, histamine signaling was determined to be cross-linked to varied intracellular signaling pathways, such as MAPK, PI3K/Akt, and NF-κB [18,19,20], which may further control the production of these inflammatory factors.

**Figure 3:**
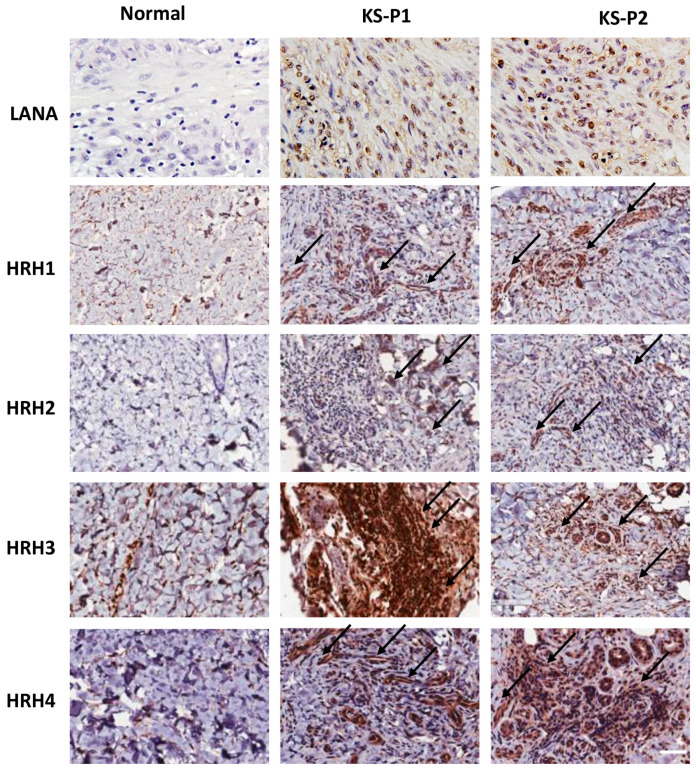
The upregulation of histamine receptor expression within AIDS-KS tissues. Expression of viral latent protein LANA and histamine receptors H1-H4 (HRH1-HRH4) in AIDS-KS tissues and normal skin tissues were determined and compared by using immunohistochemical (IHC) staining as described in the Methods section (the magnification at ×40). The arrows indicate representative HRHs-positive KS tumor cells. Bars: 50 μm.

Histamine receptors H1-H4 (HRH1-HRH4) are a class of G protein-coupled receptors (GPCRs) that bind to histamine [21]. Many studies showed that the transduction activities induced by histamine receptors include a variety of intracellular signaling pathways [18]. Interestingly, the expression of histamine receptors has been detected in many tumor cells, making them sensitive to histamine stimulation and contributing to tumor development [6]. Here, we compared the expression of different histamine receptors between normal skin tissues and AIDS-KS tissues by using IHC staining. First, the abundant expression of viral major latent protein, LANA, as the marker of KSHV latent infection in host cells, was only detected in AIDS-KS tissues. Next, our results showed that the expression of HRH1-HRH4, especially HRH1, HRH3 and HRH4, was dramatically upregulated in AIDS-KS tissues from two cancer patients when compared to those in normal skin tissues (Figure 3). In particular, many of these histamine receptors strongly positive cells are spindle cells (the typical KS tumor cells), indicating that they are potentially involved in KS progression.

**Figure 4:**
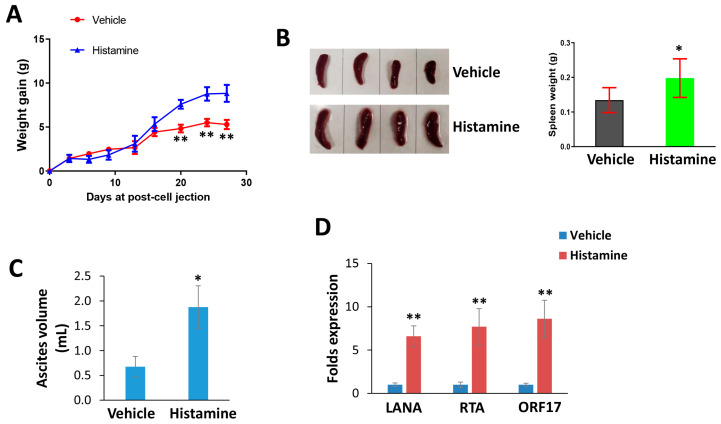
Histamine treatment promotes KSHV-infected lymphoma progression in vivo. (**A**–**C**) NOD/SCID mice were injected i.p. with BCBL-1 cells. Then, 72 h later, histamine (1.0 mg/kg) or vehicle were administered i.p. as described in the Methods section, and weights were recorded weekly. At the end of the treatment period, the ascites and spleens were collected from the histamine- or vehicle-treated mice for comparison. (**D**) The gene transcripts in ascites were quantified by using RT-qPCR. * = *p* < 0.05; ** = *p* < 0.01 (vs. the vehicle groups).

By using an established PEL xenograft model, we assessed the impacts of histamine treatment on KSHV+ lymphoma progression in vivo. We determined that histamine treatment significantly promoted PEL growth in mice, especially when ascites formation began in mice (Figure 4A). At the end of experiments, the spleens from histamine-treated mice were bigger and heavier than those from vehicle-treated mice (Figure 4B).

Moreover, histamine-treated mice had more prominent ascites formation than vehicle-treated mice (Figure 4C). We also determined that histamine treatment induced viral lytic gene expression (e.g., RTA and ORF17) in ascites cells when compared to the vehicle group (Figure 4D), which is consistent with what we observed in KSHV-infected cells during exposure to histamine treatment in vitro [9].

## 4. Discussion

In the current study, we showed that histamine treatment increased the proliferation and anchorage-independent growth of KSHV+ tumor cells, as well as tumor progression in mice models. In accordance with our findings, histamine was defined as an autocrine growth factor in experimental mammary carcinomas, potentially through certain H2 membrane receptors [22]. In addition, histamine was reported to enhance the proliferation of non-small cell lung cancer (NSCLC) cells. Histamine proliferating activity was mediated via H1, H2 and H4 receptors, and related to increased MAPK/ERK signaling activity [23].

As a global regulator in the inflammatory response, we determined that histamine treatment induced several host inflammatory factors such as IL-6, CXCL1, IFN-β and some ISGs from KSHV-infected cells. IL-6 was detected in the tumor microenvironment and peripheral circulation of patients with KSHV-related malignancies [24,25]. One recent study reported that CXCL1 production was required for the survival of KSHV-infected endothelial cells through activation of STAT3 [26]. Recent studies revealed the complex interplay between ISGs and human herpesviruses (including KSHV), and the expression profile of these ISGs varies depending on the virus, playing their antiviral or even proviral effects [27]. Therefore, these data together with our findings indicate that histamine may act as a central mediator to create a niche suitable for the survival and proliferation of KSHV-infected cells as well as virus-induced tumor development.

We determined that the expression of histamine receptors H1–H4 was all upregulated in AIDS-KS tissues when compared to those from normal skin tissues. As mentioned above, KS has different pathologic stages, such as patch, plaque and nodule. Interestingly, our previous study demonstrated that advanced KS lesions displayed higher nuclear viral LANA expression (or viral loads) [12]. Therefore, it will be interesting to compare them and determine whether the expression of these histamine receptors is differential among pathologic stages of KS. Furthermore, the functional roles of histamine receptors and downstream signaling in KS development still require more investigations.

In conclusion, our data together indicate that histamine and related signaling play important roles during KSHV infection and disease progression, which may represent attractive therapeutic targets.

## Data Availability

All the data shown in this paper are available from the corresponding authors upon reasonable request.

## Supplementary Materials

The following supporting information can be downloaded at: https://www.mdpi.com/article/10.3390/v15041011/s1, Table S1: Primer sequences for RT-qPCR.

## References

[1] Chang Y., Cesarman E., Pessin M.S., Lee F., Culpepper J., Knowles D.M., Moore P.S. (1994). Identification of herpesvirus-like DNA sequences in AIDS-associated Kaposi’s sarcoma. Science.

[2] Dupin N. (2020). Update on oncogenesis and therapy for Kaposi sarcoma. Curr. Opin. Oncol..

[3] Jenkins F.J., Hoffman L.J., Liegey-Dougall A. (2002). Reactivation of and Primary Infection with Human Herpesvirus 8 among Solid-Organ Transplant Recipients. J. Infect. Dis..

[4] Luppi M., Barozzi P., Santagostino G., Trovato R., Schulz T.F., Marasca R., Bottalico D., Bignardi L., Torelli G. (2000). Molecular evidence of organ-related transmission of Kaposi sarcoma-associated herpesvirus or human herpesvirus-8 in transplant patients. Blood.

[5] Ariza-Heredia E.J., Razonable R.R. (2011). Human Herpes Virus 8 in Solid Organ Transplantation. Transplantation.

[6] Faustino-Rocha A.I., Gama A., Neuparth M.J., Oliveira P.A., Ferreira R., Ginja M. (2017). Mast Cells in Mammary Carcinogenesis: Host or Tumor Supporters?. Anticancer Res..

[7] Xie H., He S.H. (2005). Roles of histamine and its receptors in allergic and inflammatory bowel diseases. World J. Gastroenterol..

[8] Ayers L.W., Barbachano-Guerrero A., McAllister S.C., Ritchie J.A., Asiago-Reddy E., Bartlett L.C., Cesarman E., Wang D., Rochford R., Martin J.N. (2018). Mast Cell Activation and KSHV Infection in Kaposi Sarcoma. Clin. Cancer Res..

[9] Chen J., Dai L., Goldstein A., Zhang H., Tang W., Forrest J.C., Post S.R., Chen X., Qin Z. (2019). Identification of new antiviral agents against Kaposi’s sarcoma-associated herpesvirus (KSHV) by high-throughput drug screening reveals the role of histamine-related signaling in promoting viral lytic reactivation. PLoS Pathog..

[10] Myoung J., Ganem D. (2011). Generation of a doxycycline-inducible KSHV producer cell line of endothelial origin: Maintenance of tight latency with efficient reactivation upon induction. J. Virol. Methods.

[11] An F.Q., Folarin H.M., Compitello N., Roth J., Gerson S.L., McCrae K.R., Fakhari F.D., Dittmer D.P., Renne R. (2006). Long-term-infected telomerase-immortalized endothelial cells: A model for Kaposi’s sarcoma-associated herpesvirus latency in vitro and in vivo. J. Virol..

[12] Qin Z., Freitas E., Sullivan R., Mohan S., Bacelieri R., Branch D., Romano M., Kearney P., Oates J., Plaisance K. (2010). Upregulation of xCT by KSHV-Encoded microRNAs Facilitates KSHV Dissemination and Persistence in an Environment of Oxidative Stress. PLoS Pathog..

[13] Dai L., Del Valle L., Miley W., Whitby D., Ochoa A.C., Flemington E.K., Qin Z. (2018). Transactivation of human endogenous retrovirus K (HERV-K) by KSHV promotes Kaposi’s sarcoma development. Oncogene.

[14] Dai L., Trillo-Tinoco J., Cao Y., Bonstaff K., Doyle L., Del Valle L., Whitby D., Parsons C., Reiss K., Zabaleta J. (2015). Targeting HGF/c-MET induces cell cycle arrest, DNA damage, and apoptosis for primary effusion lymphoma. Blood.

[15] Dai L., Cao Y., Jiang W., Zabaleta J., Liu Z., Qiao J., Qin Z. (2017). KSHV co-infection down-regulates HPV16 E6 and E7 from cervical cancer cells. Oncotarget.

[16] Barrett L., Chen J., Dai L., Plaisance-Bonstaff K., Del Valle L., Qin Z. (2020). Role of Interleukin-1 Family Members and Signaling Pathways in KSHV Pathogenesis. Front. Cell. Infect. Microbiol..

[17] Wei X., Lan K. (2018). Activation and counteraction of antiviral innate immunity by KSHV: An update. Sci. Bull..

[18] Dong H., Zhang W., Zeng X., Hu G., Zhang H., He S., Zhang S. (2014). Histamine Induces Upregulated Expression of Histamine Receptors and Increases Release of Inflammatory Mediators from Microglia. Mol. Neurobiol..

[19] Bakker R.A., Schoonus S.B., Smit M.J., Timmerman H., Leurs R. (2001). Histamine H(1)-receptor activation of nuclear factor-kappa B: Roles for G beta gamma- and G alpha(q/11)-subunits in constitutive and agonist-mediated signaling. Mol. Pharmacol..

[20] Nizamutdinova I.T., Dusio G.F., Gasheva O.Y., Skoog H., Tobin R., Peddaboina C., Meininger C.J., Zawieja D.C., Newell-Rogers M.K., Gashev A.A. (2016). Mast cells and histamine are triggering the NF-kappaB-mediated reactions of adult and aged perilymphatic mesenteric tissues to acute inflammation. Aging.

[21] Seifert R., Strasser A., Schneider E.H., Neumann D., Dove S., Buschauer A. (2013). Molecular and cellular analysis of human histamine receptor subtypes. Trends Pharmacol. Sci..

[22] Cricco G.P., Davio C.A., Martin G., Engel N., Fitzsimons C.P., Bergoc R.M., Rivera E.S. (1994). Histamine as an autocrine growth factor in experimental mammary carcinomas. Agents Actions.

[23] Stoyanov E., Uddin M., Mankuta D., Dubinett S.M., Levi-Schaffer F. (2012). Mast cells and histamine enhance the proliferation of non-small cell lung cancer cells. Lung Cancer.

[24] Aoki Y., Yarchoan R., Braun J., Iwamoto A., Tosato G. (2000). Viral and cellular cytokines in AIDS-related malignant lymphomatous effusions. Blood.

[25] Oksenhendler E., Carcelain G., Aoki Y., Boulanger E., Maillard A., Clauvel J.P., Agbalika F. (2000). High levels of human herpesvirus 8 viral load, human interleukin-6, interleukin-10, and C reactive protein correlate with exacerbation of multicentric castleman disease in HIV-infected patients. Blood.

[26] Lee M.J., Lee J., Kang S.K., Wirth D., Yoo S.M., Park C., Lee M.S. (2023). CXCL1 confers a survival advantage in Kaposi’s sarcoma-associated herpesvirus-infected human endothelial cells through STAT3 phosphorylation. J. Med. Virol..

[27] Gonzalez-Perez A.C., Stempel M., Chan B., Brinkmann M.M. (2020). One Step Ahead: Herpesviruses Light the Way to Understanding Interferon-Stimulated Genes (ISGs). Front. Microbiol..

