# Supplementary material for: Role of Histamine and Related Signaling in Kaposi’s Sarcoma-Associated Herpesvirus Pathogenesis and Oncogenesis
Source: Viruses. 2023 Apr 20;15(4):1011. doi: 10.3390/v15041011 (PMC10142667; doi:10.3390/v15041011)
Supplement: Supplementary file 1 [file viruses-15-01011-s001.zip › viruses-2325240-supplementary.pdf]

Supplemental Table S1. Primer sequences for RT-qPCR.

Gene	Sequences (5' ⟶ 3')
IL6	*sense GTCCAGTTGCCTTCTCCC*
	antisense GCCTCTTTGCTGCTTTCA
IL8	*sense TGGCAGCCTTCCTGATTT*
	antisense AACCCTCTGCACCCAGTT
CXCL1	*sense CACTGCTGCTCCTGCTCCT*
	antisense GGCTATGACTTCGGTTTGG
IFN-β	*sense CAAGTGTCTCCTCCAAAT*
	antisense CCTCAGGGATGTCAAAGT
IFIT1	*sense CACCCACTTCTGTCTTACT*
	antisense ACATTCTTGCCAGGTCTA
IFI44	*sense CCTGCCGTTTATTCTGTG*
	antisense CGTTTACCAACTCCCTTC
LANA	*sense TCCCTCTACACTAAACCCAATA*
	antisense TTGCTAATCTCGTTGTCCC
RTA	*sense CACAAAAATGGCGCAAGATGA*
	antisense TGGTAGAGTTGGGCCTTCAGTT
ORF17	*sense AGATTTTTCACGGGGGCTCTGG*
	antisense TGGGCTGGACACTGGGTCTATTTC
β-actin	*sense GGAAATCGTGCGTGACATT*
	antisense GACTCGTCATACTCCTGCTTG
